# miR-9, miR-21, miR-27b, and miR-34a Expression in HPV16/58/52-Infected Cervical Cancer

**DOI:** 10.1155/2020/2474235

**Published:** 2020-09-16

**Authors:** Mi Liu, Wei Wang, Haixing Chen, Yi Lu, Daisha Yuan, Yongjiu Deng, Danlu Ran

**Affiliations:** ^1^Department of Clinical Laboratory, The Tumor Hospital of Guizhou Province, Guiyang, 550004 Guizhou, China; ^2^Clinical Laboratory Medicine, Guizhou Medical University, Guiyang, 550004 Guizhou, China

## Abstract

The aim of this study was to observe the expression of miR-9, miR-21, miR-27b, and miR-34a related with E6/E7 in HPV16-, HPV52-, and HPV58-infected cervical cancer patients and explore their possible role in cervical cancer with HPV infection. The expression levels of 4 miRNAs were detected in cervical exfoliated cells using qRT-PCR. In the current study, miR-34a expression was significantly upregulated in HPV-positive cervical cancer compared with the HPV-negative healthy population and HPV-positive CIN, but just the expression of miR-34a in HPV16 cervical cancer was statistically significant, and the expression of HPV52 and HPV58 was not statistically significant. The expression of miR-21 increased in HPV-positive cervical cancer compared with HPV-positive CIN, but only HPV16-infected cervical cancer had statistical significance compared with HPV16-infected CIN. By observing the change trend of each subtype group, we can show that the expression of miR-9 in HPV16 CIN was opposite to the other subtypes, and it was upregulated, compared with HPV58 CIN, and significantly increased. The level change of miR-27b in HPV58 cervical cancer and HPV58 CIN was opposite to the other subtypes; unlike the expression of miR-27b which was upregulated in HPV16 and HPV52 infected, it was downregulated compared with Normal. We also found that the expression of miR-34a and miR-9 was contrary to other studies. These findings indicate that the upregulated miR-21 expression may be a biomarker to distinguish between CC and CIN. miR-34a in HPV infection, especially in HPV16 infection, might be related to the occurrence and development of cervical cancer. The infection of different subtypes may play different roles in disease by activating different mechanisms; miRNAs play a very complex role in tumorigenesis and development, and there may be multiple targets in which multiple mechanisms play a role.

## 1. Introduction

Cervical cancer (CC) is one of the most malignant tumors in the world, with an estimated 570,000 new cases and 311,000 deaths of cervical cancer in 2018 worldwide [1]. As the China National Cancer Center published, cervical cancer ranked 6th (6.25%) and 8th (3.96%), respectively, in female cancer incidence and mortality in China in 2015 [2].

Persistent infection by high-risk human papilloma virus (HR-HPV) is considered the major factor in the development of cervical cancer and can be detected in 99.7% of cervical squamous cell carcinomas [3]. The genetic instability and the overall destruction of cell gene expression caused by the abnormal expression of HPV E6 and E7 oncoproteins are the important reasons in carcinogenesis [4, 5]. The protein encoded by E6 and E7 can regulate the growth and replication of the virus and has the function of cell transformation. E6 and E7 bind to the tumor suppressive p53 and pRb, respectively, which leads to the inactivation of p53 and pRb, destroys the normal regulation of cell proliferation cycle, interferes with the function of p53 and pRb in inhibiting cell division and proliferation, and finally leads to the immortalization and sustained malignant proliferation of cells. Recent studies have shown that abnormal increase of E6 and E7 can regulate the transcriptional or posttranscriptional of microRNAs (miRNA) [6–8]. Therefore, to study miRNA related with E6 and E7, we can choose some potential therapeutic target and biomarkers for cervical cancer.

Although HPV16 and HPV18 are the main subtypes that caused cervical cancer in many countries and regions [9], there are regional and ethnic differences in the distribution of HPV subtypes infected with cervical cancer. HPV16, HPV18, HPV33, HPV58, and HPV52 are the major oncogenic genotypes of HPV-related cervical cancer in Asia [10–12]. HPV35, HPV16, and HPV31 are the major oncogenic genotypes in Africa [13]. HPV16, HPV31, and HPV18 are the most important cervical cancer-related genotypes in Europe [14], while in South America they are HPV16 and HPV58 [15]. Studies have shown that the expression levels of miRNA may be different with different HPV subtypes in cervical cancer. Martinez et al. [6] compared the miRNA expression between HPV16-positive cervical cancer cell line and HPV-negative cell line; 24 miRNAs were downregulated, and 3 miRNAs were upregulated. The HPV18-positive cell line had 14 downregulated miRNAs, 6 of which were different from the HPV16-positive cell line; 13 miRNAs were upregulated, 11 of which were different from the HPV16-positive cell line. Gunasekharan and his colleagues analyzed the HPV31-transfected human foreskin keratinocytes and normal cells from the same donor and found that the expression of 55 kinds of miRNAs was inhibited and 38 kinds of miRNAs were increased [7].

The Guizhou Province of China is a multiethnic gathering community with mountainous plateau and humid climate. The common subtypes of cervical cancer infected by HPV are different from other regions. In the previous study of our team, we found that HPV subtypes of cervical cancer patients ranked in the top three are HPV16, HPV52, and HPV58 in the Tumor Hospital of Guizhou Province [16], similar to those in southern China and different from those in northern China, Europe, and the United States. The aim of this study was to observe the expression of four miRNAs (miR-9, miR-21, miR-27b, and miR-34a) related with E6/E7 in HPV16-, HPV52-, and HPV58-infected cervical cancer patients and explore their possible role in cervical cancer with HPV infection.

## 2. Materials and Method

### 2.1. Patients and Sample

116 cervical exfoliated cells from patients with HPV16, HPV52, and HPV58 infection were collected as the study population in the Tumor Hospital of Guizhou Province from December 2016 to March 2019, including 75 cervical cancer (CC) patients (mean age 50.2 ± 10.1) and 41 cervical intraepithelial neoplasia (CIN) patients (mean age 44.6 ± 9.9), grouping according to pathological diagnosis and HPV genotyping, and untreated at the time of the study. At the same time, 33 cervical exfoliated cells from HPV-negative healthy people were collected as control (Normal) (mean age 42.7 ± 7.1). Samples were collected in cervical exfoliated cell preservation solution (Kaipu Biotechnology Co., Guangdong, China). All the clinical samples were immediately frozen and stored at −80°C for further studies. This study was approved by the ethics committee of the Tumor Hospital of Guizhou Province, and exemption of informed consent was applied according to the technical guidelines of clinical trials of in vitro diagnostic reagents in China.

### 2.2. HPV Typing

HPV genotypes were detected by flow through hybridization and low-density microarray (Hybribio, Chaozhou, China). The test can detect 21 types, including 15 HR-HPV types: 16, 18, 31, 33, 35, 39, 45, 51, 52, 53, 56, 58, 59, 66, and 68; and 5 low-risk types: 6, 11, 42, 43, 44, and cp8304 (81).

### 2.3. RNA Extraction and Quantitative Real-Time PCR (qRT-PCR)

Small RNAs were extracted from cervical exfoliated cells using RNAiso Plus reagent (Takara, Shiga, Japan). Small RNA was reversed transcribed to cDNA using Mir-X miRNA First-Strand Synthesis Kit (Clontech, Takara, Japan). Real-time PCR for miRNA expression was performed using SYBR green I mix (Clontech, Takara, Japan), and U6 was used as an endogenous control to normalize miRNA expression. All tests followed the manufacturer's instruction. The specific primers for miR-9 (miR-9-5P), miR-21 (miR-21-5P), miR-27b (miR-27b-5P), and miR-34a (miR-34a-5P) are from GeneCopoeia (Guangzhou, China). The relative expression levels of each sample were measured using the 2^−*ΔΔ*Ct^ method and normalized to a control (2^−ΔΔCt^ = 2^–[(miRNAs Ct of experimental group–U6 Ct of experimental group) − (miRNAs Ct of control group − U6 Ct of control group)]^). All qPCR assays were performed on an Applied Biosystems 7500 system (Applied Biosystems, Warrington, UK).

### 2.4. Statistical Analysis

All data were expressed as mean ± standard deviation (SD). Statistical analysis was performed using IBM SPSS 20.0 (SPSS, Chicago, IL, USA), and the GraphPad Prism 6 (GraphPad Software, San Diego, CA, USA) was used to draw a graph. The comparison between groups was detected by two independent samples *t*-test, and a value of *P* < 0.05 was considered statistically significant.

## 3. Results

### 3.1. Expression of Four miRNAs in HPV-Infected Cervical Cancer

**Figure 1:**
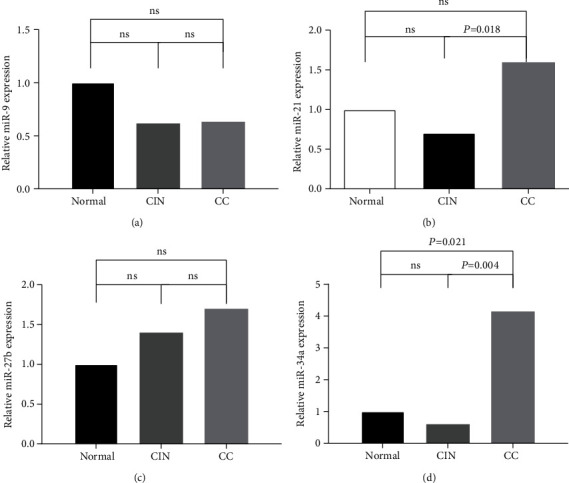
Expression of four E6/E7-related miRNAs in CC and CIN. (a) Relative miR-9 expression in CIN and CC. (b) Relative miR-21 expression in CIN and CC. (c) Relative miR-27b expression in CIN and CC. (d) Relative miR-34a expression in CIN and CC. ns: the difference was not statistically significant.

Compared with Normal, the expression of miR-9 in CC and CIN decreased, but the difference was not statistically significant (*P* > 0.05) (Figure 1(a)). Compared with Normal, the expression of miR-21 increased in CC and decreased in CIN, but the difference was not statistically significant (*P* > 0.05) (Figure 1(b)); further comparison between CC and CIN of miR-21 showed that the expression in CC significantly increased compared with CIN (2.257-fold, *P* = 0.018) (Figure 1(b)). The expression of miR-27b in CC and CIN increased compared with Normal, but the difference was not statistically significant (*P* > 0.05) (Figure 1(c)). The expression of miR-34a in CC was upregulated compared with CIN and Normal (4.141- and 2.466-fold, *P* = 0.004 and 0.021, resp.) (Figure 1(c)). The results are shown in Table 1 and Figure 1.

### 3.2. Expression of Four miRNAs in Cervical Cancer Infected with Three Different Subtypes of HPV

**Figure 2:**
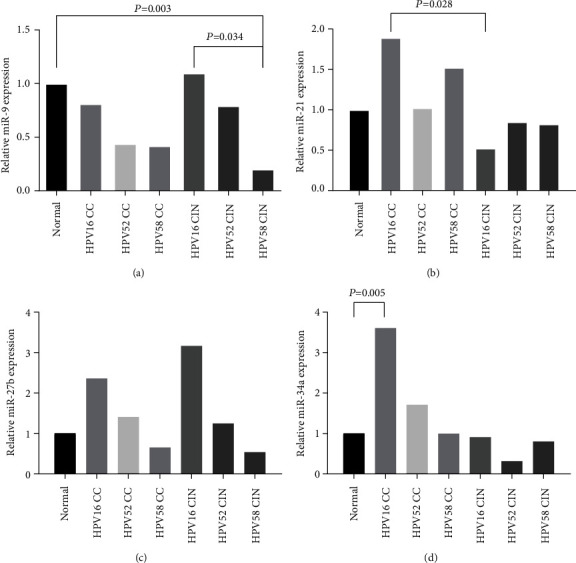
Expression of four E6/E7-related miRNAs in different HPV subtypes. (a) Relative miR-9 expression in HPV subtype groups. (b) Relative miR-21 expression in HPV subtype groups. (c) Relative miR-27b expression in HPV subtype groups. (d) Relative miR-34a expression in CIN and CC. ns: the difference was not statistically significant.

In order to understand whether there are differences in 4 miRNA expression in HPV16-, HPV52-, and HPV58-infected cervical cancer, depending on the type of infection, 75 cervical cancer patients were divided into HPV16 CC (*n* = 44, mean age 49.5 ± 9.6), HPV52 CC (*n* = 16, mean age 48.4 ± 9.0), and HPV58 CC (*n* = 12, mean age 54.8 ± 12.6). Compared with Normal, except for the expression of miR-34a in HPV16 CC that increased (3.609-fold, *P* = 0.005) (Figure 2(d)), the expression of other miRNAs in 3 sorts of HPV-infected cervical cancer was not statistically significant; there was no significant difference in the expression of 4 miRNAs among the 3 subtypes. By observing the change trend of each subtype group, we can show that most of the subtype groups and CC were similar, but the change trend of miR-27b in HPV58 CC was opposite to the overall trend, showing a downregulation compared with Normal, despite not being statistically significant.

41 CIN patients were divided into HPV16 CIN (*n* = 15, mean age 41.9 ± 10.6), HPV52 CIN (*n* = 15, mean age 49.5 ± 8.8), and HPV58 CIN (*n* = 11, mean age 41.6 ± 8.5). The expression of miR-9 in HPV58 CIN significantly decreased compared with Normal (0.020-fold, *P* = 0.003) (Figure 2(a)). The change trend of HPV16 CIN was opposite to the whole, and its expression was upregulated, and compared with HPV58 CIN, it significantly increased (5.476-fold, *P* = 0.034) (Figure 2(a)). The expression of miR-21 decreased in HPV16, HPV52, and HPV58 CIN, which were consistent with the overall trend, but there was no significant difference. But the expression of miR-21 in HPV16 CC was 3.612-fold higher than HPV16 CIN (*P* = 0.028) (Figure 2(b)). There was no significant difference in the expression of miR-27b in the three subtype groups compared with Normal. However, similar to the HPV58 CC, the change trend of miR-27b in the HPV58 CIN was opposite from the overall trend, which showed downregulation compared with Normal. The expression of miR-34a in the three subtype groups showed a downward trend compared with Normal, but the difference was not statistically significant. The results are shown in Figure 2 and Table 2.

## 4. Discussion

Cervical cancer is one of the malignancies that threaten the health of women, and early diagnosis and therapy may improve the survival rate. HPV-DNA typing is one of the indicators for cervical cancer screening; however, this test just shows whether there is HPV infection and cannot be used for the diagnosis of cervical cancer. Therefore, it is valuable to search some potential therapeutic targets and biomarkers for cervical cancer.

HR-HPV infection is the main cause of cervical cancer. It has been shown that the integration of the viral genome into the host chromosome may reflect E6/E7-mediated genomic instability. E6 can lead to the ubiquitination of tumor suppressor p53 and the degradation of PDZ [17]. The combination of E7 and pRb may inhibit E2F activity and lose antitumor effect [18]. These complex interactions between E6/E7 and transcription factor can induce the expression of miRNA, and miRNA binds to its mRNA targets with a high complementarity, regulating the protein function, thus affecting the occurrence, development, metastasis, and prognosis of HPV-related cervical cancer [19]. Multiple studies have shown that miRNA expression is different in cervical cancer tissues and cells, considering it a potential therapeutic target and biomarker of cervical cancer [20, 21].

There are many subtypes of HPV, the main infection subtypes in different regions are different, and the expression of miRNAs in different subtypes is also different. Four miRNAs (miR-9, miR-21, mir-27b, and miR-34a) that are related to E6/E7 were selected, and their expression was analyzed in cervical cancer patients infected with HPV16, HPV52, and HPV58, which are the most common HPV subtypes in cervical cancer patients in the Tumor Hospital of Guizhou Province, and their possible role in cervical cancer with HPV infection was explored.

The abnormal expression of miR-9 plays an important role in the occurrence and development of cervical cancer by affecting the metabolism of tumor cells such as ATPase activity, radical group transfer enzyme metabolism, and glutamine amino acid metabolism [22]. The activity of miR-9 was related to the transcription of HPV E6/E7; they could induce the activation of miR-9 which led to the significant increase of cell migration by upregulating multiple gene targets [23]. Studies showed that miR-9 expression significantly increased in HPV-positive cervical cancer tissues [24]. However, some studies have shown opposite results. Wan and Zhang's [25] research pointed out that the content of miR-9 in HPV-positive cervical cancer was significantly downregulated than that in HPV-negative cervical cancer patients and analyzed that miR-9 was targeted at the protooncogene; when the level of miR-9 targeted at the protooncogene decreased, it would lead to the increase of protooncogene expression and then cause the occurrence and development of cervical cancer. The study showed that the expression of miR-9 in HPV-positive cervical cancer and CIN decreased compared with the HPV-negative healthy population, and the change trend was similar to that of Wan and Zhang, though the difference was not statistically significant. The expression of miR-9 in HPV58-infected CIN also significantly decreased compared with that in HPV-negative healthy population. Unlike HPV52 and HPV58, the expression of HPV16-infected CIN was upregulated, which was significantly higher than that of HPV58-infected CIN. The results suggest that the expression of miR-9 was different in different HPV subtypes of infection, and their roles may be different in different subtype-infected cervical diseases. When HPV58 infects the cervical cells, it may promote the occurrence and development of CIN by downregulating miR-9.

miR-21 is a tumor growth regulator, which plays an important role in tumor angiogenesis, invasion, and metastasis. It is highly expressed in many cancers and is considered to have a carcinogenic effect [26, 27]. Yao and Lin [28] reported that miR-21 was upregulated in cervical cancer patients with HPV infection, suggesting that HPV infection induces carcinogenesis probably through altering expression of some oncomiRs such as miR-21; miR-21 could be used as an oncogene in cervical cancer, and HPV16 E6 and E7 oncoproteins are involved in this process. The study showed that the expression of miR-21 was upregulated in HPV-positive cervical cancer, and the change trend was consistent with the above study, but the difference was not statistically significant. Unlike with cervical cancer, miR-21 expression was downregulated in HPV-positive CIN. The miR-21 expression in HPV-positive CC increased 2.266-fold compared with that in CIN. Also, the miR-21 expression in HPV16-positive CC increased 3.618-fold compared with that in HPV16-positive CIN. Although the expression of miR-21 in HPV52- and HPV58-infected cervical cancer was also higher than that in CIN infected with the same type, the difference was not statistically significant. The results indicate that the expression of miR-21 is different in cervical cancer and CIN, which may be a biomarker to distinguish cervical cancer and CIN, especially in HPV16-infected cervical cancer, and miR-21 may play an important role.

miR-27 family can be divided into two subtypes: miR-27a and miR-27b. miR-27b can regulate the budding and proliferation of endothelial cells in vitro and promote the angiogenesis in vivo. For the role of miR-27b in tumor, different studies show different results. Some studies have shown that miR-27b inhibits tumor progression and angiogenesis in colorectal cancer by targeting VEGFC. Other studies showed that miR-27b could promote the proliferation and invasion of breast cancer cells by inhibiting the expression of ST-14. miR-27b upregulated by HPV16 E7 promotes proliferation and suppresses apoptosis by targeting polo-like kinase2 in cervical cancer [29]. In this study, the expression of miR-27b in HPV-positive cervical cancer was higher than that in HPV-positive CIN and HPV-negative healthy population, but the difference was not statistically significant. Interestingly, the expression of miR-27b of HPV58-infected cervical cancer and CIN was downregulated, which was different from other subtypes. It indicated that the role of miR-27b in HPV58-infected cervical diseases may be different from other subtypes and deserves further study.

miR-34a is a classical tumor suppressor gene [30, 31]. The expression of miR-34a can be significantly increased by activating p53 pathways and then play a role in inhibiting tumor [32, 33]. Many studies have confirmed the decrease of miR-34a expression in HPV-infected cervical cancer tissue and cancer cells [34, 35]. Researchers found the decreased expression of miR-34a in HPV16+ cell compared with HPV18+ cell and HPV-negative cell, used HPV18+ HeLa cells to verify the relationship between E6/E7 and miR-34a and p53, and found that the cells expressing E7 had S-phase interruption, and only the tumor suppressor gene expressed in E6 cells was inhibited. Their study showed that abnormal expression of E6 in cervical cancer would inhibit miR-34a expression and then inhibit the expression of p53 and promote the development of tumor [36, 37]. However, Ribeiro et al. [38] showed the opposite results. They tested the cervical cells of patients and showed that the expression of miR-34a was significantly higher than that of uninfected people, which was contrary to the results of most cell lines. Our study showed that the expression of miR-34a in HPV-positive cervical cancer patients was significantly upregulated than that in HPV-positive CIN patients and HPV-negative healthy population (2.930- and 2.987-fold, resp.), and the expression of miR-34a in HPV16+ cervical cancer patients also significantly increased than that in HPV16+ CIN patients. The results were similar to those of Ribeiro et al. It is suggested that miR-34a may have other mechanisms in the occurrence and development of cervical cancer and its expression level may change in different stages of the disease. Some researchers believe that the inhibition of miR-34a expression may be an early event in the development of cervical cancer [39]. With the development of the disease, some other pathways, such as the cellular repair mechanisms, may be activated after viral infection, which would activate p53 pathways and induce the expression of miR-34a [38]. Therefore, it is worth performing more studies on miR-34a levels and finding out other mechanisms besides the p53-associated pathways, which are able to affect the level change of miR-34a in HPV-infected cervical cancer, especially in HPV16 infected.

In conclusion, our study showed that the expression of some miRNAs was abnormal in HPV-positive cervical cancer. The upregulated expression of miR-21 may be a biomarker to distinguish cervical cancer and CIN, while the upregulation of miR-34a in HPV infected, especially in HPV16 infected, may be related to the occurrence and development of cervical cancer. Although there was no significant difference in the expression of four miRNAs in cervical cancer patients with different HPV subtypes, the trend of the miRNA levels in different subtypes is not the same. The expression of miR-9 in HPV16 CIN was opposite to the other subtype, and it was upregulated; compared with HPV58 CIN, it significantly increased. The expression of miR-27b decreased in HPV58, while it increased in HPV16- and HPV52-infected cervical cancer. It was suggesting that infection of different subtypes may play different roles in disease by activating different mechanisms. We also found that the expression of miR-34a is significantly upregulated and miR-9 is downregulated in HPV-positive cervical cancer, which was contrary to other studies. It shows that miRNAs play a very complex role in tumorigenesis and development, and there may be multiple targets in which multiple mechanisms play a role. Therefore, there is still a lot of research work to be done in the selection of potential therapeutic target and clinical biomarkers.

## Figures and Tables

**Table 1:** Quantitative real-time PCR (qRT-PCR) data analysis of four E6/E7-related miRNAs.

	*n*	miR-9	miR-21	miR-27b	miR-34a
Compared with Normal			2^–*ΔΔ*Ct^ (*P* value)		
CC	75	0.632 (0.248)	1.593 (0.238)	1.698 (0.238)	2.466 (0.021)^∗^
CIN	41	0.610 (0.237)	0.706 (0.313)	1.397 (0.526)	0.596 (0.251)
Compared with CIN					
CC	33	1.026 (0.957)	2.257 (0.018)^∗^	1.216 (0.677)	4.141 (0.004)^∗∗^

^∗^ *P* < 0.05; ^∗∗^*P* < 0.01.

**Table 2:** qRT-PCR data analysis of E6/E7-related miRNAs in different HPV subtypes.

	miR-9	miR-21	miR-27b	miR-34a
Compared with Normal	2^–*ΔΔ*Ct^ (*P* value)			
HPV16 CC (*n* = 44)	0.809 (0.668)	1.892 (0.106)	2.360 (0.072)	3.609 (0.005)^∗∗^
HPV52 CC (*n* = 16)	0.438 (0.233)	1.025 (0.964)	1.409 (0.586)	1.708 (0.502)
HPV58 CC (*n* = 12)	0.418 (0.096)	1.523 (0.448)	0.651 (0.515)	0.996 (0.993)
HPV16 CIN (*n* = 15)	1.096 (0.860)	0.524 (0.194)	3.166 (0.076)	0.907 (0.893)
HPV52 CIN (*n* = 15)	0.792 (0.630)	0.850 (0.692)	1.246 (0.721)	0.315 (0.133)
HPV58 CIN (*n* = 11)	0.200 (0.003)^∗∗^	0.822 (0.689)	0.534 (0.563)	0.801 (0.662)
Comparison between groups				
HPV16 CC vs. HPV52 CC	1.848 (0.444)	1.847 (0.305)	1.675 (0.433)	2.113 (0.323)
HPV16 CC vs. HPV58 CC	1.937 (0.443)	1.242 (0.735)	3.623 (0.074)	3.625 (0.099)
HPV52 CC vs. HPV58 CC	1.048 (0.956)	0.673 (0.656)	2.163 (0.399)	1.716 (0.596)
HPV16 CIN vs. HPV52 CIN	1.384 (0.648)	0.616 (0.413)	2.541 (0.276)	2.883 (0.283)
HPV16 CIN vs. HPV58 CIN	5.476 (0.034)^∗^	0.637 (0.524)	5.926 (0.125)	1.132 (0.891)
HPV52 CIN vs. HPV58 CIN	3.957 (0.054)	1.034 (0.948)	2.333 (0.441)	0.393 (0.315)
HPV16 CC vs. HPV16 CIN	0.739 (0.703)	3.612 (0.028)^∗^	0.745 (0.661)	3.979 (0.067)
HPV52 CC vs. HPV52 CIN	0.553 (0.454)	1.206 (0.785)	1.130 (0.884)	5.429 (0.108)
HPV58 CC vs. HPV58 CIN	2.088 (0.309)	1.854 (0.440)	1.219 (0.866)	1.243 (0.784)

^∗^ *P* < 0.05; ^∗∗^*P* < 0.01.

## Data Availability

The data used to support the findings of this study are available from the corresponding author upon request.

## References

[1] Bray F., Ferlay J., Soerjomataram I., Siegel R. L., Torre L. A., Jemal A. (2018). Global cancer statistics 2018: GLOBOCAN estimates of incidence and mortality worldwide for 36 cancers in 185 countries. *CA: a Cancer Journal for Clinicians*.

[2] Zheng R. S. (2019). Report of cancer epidemiology in China, 2015. *Chinese Joural of Oncology*.

[3] Cortez M. A., Bueso-Ramos C., Ferdin J., Lopez-Berestein G., Sood A. K., Calin G. A. (2011). MicroRNAs in body fluids-the mix of hormones and biomarkers. *Nature Reviews Clinical Oncology*.

[4] Crosbie E. J., Kitchener H. C. (2006). Human papillomavirus in cervical screening and vaccination. *Clinical Science*.

[5] Jiménez-Wences H., Peralta-Zaragoza O., Fernández-Tilapa G. (2014). Human papilloma virus, DNA methylation and microRNA expression in cervical cancer (review). *Oncology Report*.

[6] Martinez I., Gardiner A. S., Board K. F., Monzon F. A., Edwards R. P., Khan S. A. (2008). Human papillomavirus type 16 reduces the expression of microRNA-218 in cervical carcinoma cells. *Oncogene*.

[7] Gunasekharan V., Hache G., Laimins L. (2012). Differentiation-dependent changes in levels of C/EBP*β* repressors and activators regulate human papillomavirus type 31 late gene expression. *Journal of Virology*.

[8] Wang X. H., Wang H. K., Li Y. (2014). MicroRNAs are biomarkers of oncogenic human papillomavirus infections. *Proceedings of the National Academy of Sciences of the United States of America*.

[9] Herrero R. (2003). Human papillomavirus and oral cancer: the International Agency for Research on Cancer Multicenter Study. *Journal of the National Cancer Institute*.

[10] Othman N. H., Rebolj M. (2009). Challenges to cervical cancer screening in a developing country: the case of Malaysia. *Asian Pacific Journal of Cancer Prevention*.

[11] Weinberg C. R. (2009). HPV screening for cervical cancer in rural India. *The New England Journal of Medicine*.

[12] Wang S. Z., Wei H., Wang N. (2012). The prevalence and role of human papillomavirus genotypes in primary cervical screening in the northeast of China. *BMC Cancer*.

[13] Peters L. M., Soliman A. S., Bukori P., Mkuchu J., Ngoma T. (2010). Evidence for the need of educational programs for cervical screening in rural Tanzania. *Journal of Cancer Education*.

[14] Liu X. Q., Li Z. C., Li Y. W. (2015). The status of HPV infection in female medical personnel in Shenzhen and the research of HPV gene typing. *Intenational Journal of Laboratory Medicine*.

[15] Luyten A., Buttmann Schweiger N., Luyten K. (2014). Early detection of CIN3 and cervical cancer during long term follow up using HPV/Pap smear co-testing and risk-adapted follow up in a locally organized screening program. *International Journal of Cancer*.

[16] Liu M., Wang W., Lu Y., Chen H. X., Zhao Q. B. (2018). Distribution of HPV subtypes in women in cancer hospital of Guizhou University, 2010-2016. *Modern Preventive Medicine*.

[17] Thomas M., Pim D., Banks L. (1999). The role of the E6-p53 interaction in the molecular pathogenesis of HPV. *Oncogene*.

[18] Duensing S., Lee L. Y., Duensing A. (2000). The human papillomavirus type 16 E6 and E7 oncoproteins cooperate to induce mitotic defects and genomic instability by uncoupling centrosome duplication from the cell division cycle. *Proceedings of the National Academy of Sciences of the United States of America*.

[19] Pedroza-Torres A., López-Urrutia E., García-Castillo V. (2014). MicroRNAs in cervical cancer: evidences for a miRNA profile deregulated by HPV and its impact on radio-resistance. *Molecules*.

[20] Fujii T., Shimada K., Asano A. (2016). MicroRNA-331-3p suppresses cervical cancer cell proliferation and E6/E7 expression by targeting NRP2. *International Journal of Molecular Sciences*.

[21] Zhang L., Zhan X., Yan D. D., Wang Z. H. (2016). Circulating microRNA-21 is involved in lymph node metastasis in cervical cancer by targeting RASA1. *International Journal of Gynecological Cancer*.

[22] Hu X., Schwarz J. K., Lewis J. S. (2010). A microRNA expression signature for cervical cancer prognosis. *Cancer Research*.

[23] Liu W., Gao G., Hu X. (2014). Activation of miR-9 by human papillomavirus in cervical cancer. *Oncotarget*.

[24] Park S., Eom K., Kim J. (2017). miR-9, miR-21, and miR-155 as potential biomarkers for HPV positive and negative cervical cancer. *BMC Cancer*.

[25] Wan S. Q., Zhang J. (2013). Study of the differences of mi RNA between HPV positive and HPV negative cervical carcinoma tissue. *Journal of Hainan Medical College*.

[26] Li X., Xin S., He Z. (2014). MicroRNA-21 (miR-21) post-transcriptionally downregulates tumor suppressor PDCD4 and promotes cell transformation, proliferation, and metastasis in renal cell carcinoma. *Cellular Physiology and Biochemistry*.

[27] Liu C. Z., Liu W., Zheng Y. (2012). PTEN and PDCD4 are bona fide targets of microRNA-21 in human cholangiocarcinoma. *Chinese Medical Sciences Journal*.

[28] Yao T., Lin Z. (2012). MiR-21 is involved in cervical squamous cell tumorigenesis and regulates CCL20. *Biochimica et Biophysica Acta*.

[29] Liu F., Zhang S. M., Zhao Z. (2016). MicroRNA-27b up-regulated by human papillomavirus 16 E7 promotes proliferation and suppresses apoptosis by targeting polo-like kinase2 in cervical cancer. *Oncotarget*.

[30] Liu C., Kelnar K., Liu B. (2011). The microRNA miR-34a inhibits prostate cancer stem cells and metastasis by directly repressing CD44. *Nature Medicine*.

[31] Hu C. E., Liu Y. C., Zhang H. D., Huang G. J. (2014). The RNA-binding protein PCBP2 facilitates gastric carcinoma growth by targeting miR-34a. *Biochemical and Biophysical Research Communications*.

[32] Yamakuchi M., Ferlito M., Lowenstein C. J. (2008). MiR-34a repression of SIRT1 regulates apoptosis. *Proceedings of the National Academy of Sciences of the United States of America*.

[33] Tazawa H., Tsuchiya N., Izumiya M., Nakagama H. (2007). Tumor-suppressive miR-34a induces senescence-like growth arrest through modulation of the E2F pathway in human colon cancer cells. *Proceedings of the National Academy of Sciences of the United States of America*.

[34] Akao Y., Noguchi S., Lio A., Kojima K., Takagi T., Naoe T. (2011). Dysregulation of microRNA-34a expression causes drug-resistance to 5-FU in human colon cancer DLD-1 cells. *Cancer Letters*.

[35] Wang X., Meyers C., Guo M., Zheng Z. M. (2011). Upregulation of p18Ink4c expression by oncogenic HPV E6 via p53-miR-34a pathway. *International Journal of Cancer*.

[36] Zhang R., Su J., Xue S. L. (2016). HPV E6/p53 mediated down-regulation of miR-34a inhibits Warburg effect through targeting LDHA in cervical cancer. *American Journal of Cancer Research*.

[37] Wang X. H., Wang H. K., McCoy J. P. (2009). Oncogenic HPV infection interrupts the expression of tumor-suppressive miR-34a through viral oncoprotein E6. *RNA*.

[38] Ribeiro J., Marinho-Dias J., Monteiro P. (2015). miR-34a and miR-125b expression in HPV infection and cervical cancer development. *BioMed Research International*.

[39] Hermeking H. (2012). MicroRNAs in the p53 network: micromanagement of tumour suppression. *Nature Reviews Cancer*.

